# Predictors of persistent symptoms and reduced quality of life in treated coeliac disease patients: a large cross-sectional study

**DOI:** 10.1186/1471-230X-13-75

**Published:** 2013-04-30

**Authors:** Pilvi Paarlahti, Kalle Kurppa, Anniina Ukkola, Pekka Collin, Heini Huhtala, Markku Mäki, Katri Kaukinen

**Affiliations:** 1 Tampere Centre for Child Health Research, University of Tampere and Tampere University Hospital, Tampere, Finland; 2 Department of Gastroenterology and Alimentary Tract Surgery, Tampere University Hospital and School of Medicine, University of Tampere, Tampere, Finland; 3 Tampere School of Health Sciences, University of Tampere, Tampere, Finland; 4 Seinäjoki Central Hospital, Seinäjoki, Finland

**Keywords:** Coeliac disease, Symptoms, Quality of life, Gluten-free diet, Adults

## Abstract

**Background:**

Evidence suggests that many coeliac disease patients suffer from persistent clinical symptoms and reduced health-related quality of life despite a strict gluten-free diet. We aimed to find predictors for these continuous health concerns in long-term treated adult coeliac patients.

**Methods:**

In a nationwide study, 596 patients filled validated Gastrointestinal Symptom Rating Scale and Psychological General Well-Being questionnaires and were interviewed regarding demographic data, clinical presentation and treatment of coeliac disease, time and place of diagnosis and presence of coeliac disease-associated or other co-morbidities. Dietary adherence was assessed by a combination of self-reported adherence and serological tests. Odds ratios and 95% confidence intervals were calculated by binary logistic regression.

**Results:**

Diagnosis at working age, long duration and severity of symptoms before diagnosis and presence of thyroidal disease, non-coeliac food intolerance or gastrointestinal co-morbidity increased the risk of persistent symptoms. Patients with extraintestinal presentation at diagnosis had fewer current symptoms than subjects with gastrointestinal manifestations. Impaired quality of life was seen in patients with long duration of symptoms before diagnosis and in those with psychiatric, neurologic or gastrointestinal co-morbidities. Patients with persistent symptoms were more likely to have reduced quality of life.

**Conclusions:**

There were a variety of factors predisposing to increased symptoms and impaired quality of life in coeliac disease. Based on our results, early diagnosis of the condition and consideration of co-morbidities may help in resolving long-lasting health problems in coeliac disease.

## Background

At present, the only treatment for coeliac disease is a lifelong gluten-free diet, i.e. exclusion of wheat-, rye- and barley-containing cereals and food products. Upon removal of gluten from the diet clinical symptoms are usually rapidly alleviated, while recovery of the duodenal mucosa may take several months or even years [1]. The treatment may also prevent many coeliac disease-associated complications such as intestinal malignancies [2]. Notwithstanding these benefits, the stigma of a chronic disorder and the need for major dietary restrictions increases the self-perceived burden of illness and may impair patients’ quality of life [3,4]. The symptoms may also remain despite a long-term and strict diet [5,6]. As a result, in some studies even well-treated coeliac patients have failed to attain well-being similar to that of the population in general [7,8], albeit that there are also contradictory results [9,10]. To improve the situation, knowledge of the factors underlying these persistent health concerns in coeliac patients is required. Thus far, only a limited number of studies have investigated this issue [11]; data are scant, particularly in screen-detected patients and in subjects with extraintestinal presentation [9,12].

The aim of this large nationwide study was to find predictors of persistent gastrointestinal symptoms and reduced health-related quality of life in long-term treated adult coeliac patients. Particular attention was devoted to aspects such as duration and severity of symptoms before diagnosis and presence of coeliac disease-associated and other co-morbidities.

## Methods

### Study design and participants

The trial was conducted at the Department of Gastroenterology and Alimentary Tract Surgery, Tampere University Hospital. Adult volunteers were recruited by a nationwide search using newspaper advertisements and via national and local coeliac disease societies. The study cohort comprised a total of 596 adults (age > 18 years) with biopsy-proven coeliac disease. All coeliac diagnoses and other relevant medical data (see below) were confirmed from the patients’ medical records. Subjects who had been on a gluten-free diet less than one year, had biopsy-proven refractory coeliac disease, or whose coeliac disease diagnosis could not be verified were excluded from the study.

All eligible subjects filled self-administered, validated and structured gastrointestinal symptom and corresponding health-related quality of life questionnaires. Next, to reveal factors associated with persistent symptoms and poor quality of life, the participants were interviewed by a physician or study nurse with expertise in coeliac disease. They were asked to report their demographic data and family history of coeliac disease, clinical presentation of the condition, time and place of diagnosis (primary, secondary or tertiary health care), dietary counseling and regular follow-up, adherence to the gluten-free diet, possible consumption of oats and duration of diet. Furthermore, the presence of coeliac disease-associated or other significant co-morbidities such as autoimmune thyroidal disease or type 1 diabetes, was inquired after.

Clinical presentation at diagnosis was further categorized into gastrointestinal (any kind of gastrointestinal symptom or signs of malabsorption), extraintestinal (e.g. dermatitis herpetiformis, neurological symptoms or arthralgia) and screen-detected (subjects identified by screening in at-risk groups). Duration of symptoms was defined as first experienced symptom until diagnosis, and was divided into three subgroups as follows: no symptoms, symptoms 10 years or less and symptoms more than 10 years. Further, the self-perceived severity of the symptoms was asked and divided into three subgroups as follows: no symptoms, moderate symptoms and severe symptoms.

Altogether 110 healthy subjects (81% females, median age 49 (range 24–87) years) having no first-degree relatives with coeliac disease were used as a non-coeliac control group. The controls were recruited from the close neighborhood and among friends of the coeliac patients.

All participants gave written informed consent. The study protocol was approved by the Ethical Committee of Tampere University Hospital.

### Health-related quality of life and gastrointestinal symptoms

The current self-perceived gastrointestinal symptoms and health-related quality of life of the participants were evaluated by structured and well-validated questionnaires widely applied in coeliac disease research [7,12-15]. The Gastrointestinal Symptom Rating Scale (GSRS) [16,17] was used to evaluate gastrointestinal symptoms. The questionnaire comprises 15 separate items covering five different sub-dimensions: diarrhoea, indigestion, constipation, abdominal pain and reflux. The scoring is based on a 7-grade Likert scale in which 1 point indicates no symptoms and 7 points the most severe gastrointestinal symptoms. Values for each sub-dimension score are calculated as a mean of the relevant items. The total GSRS score is calculated as a mean value of all 15 items and may thus also gain values between 1 to 7 points. Patients with a score higher than 1 standard deviation (SD, 0.66 points) compared to the control mean were considered to have increased gastrointestinal symptoms [18-20]. The threshold score for increased symptoms was therefore 2.55 points.

The Psychological General Well-Being (PGWB) questionnaire [12,13,15,21] was used to measure health-related quality of life. This survey consists of 22 separate items covering six different sub-dimensions: anxiety, depression, well-being, self-control, general health and vitality. The scoring is based on a 6-grade Likert scale in which higher scores indicate better quality of life. The value of the total PGWB score may range from a minimum of 22 to maximum 132. The sub-scores are calculated as a sum of the items in each sub-dimension in question. Subjects with a score less than 1 SD (12.1 points) compared to the control mean were considered to have reduced quality of life [18-20], so the threshold score was 93.1 points.

### Adherence to the gluten-free diet

The possible consumption of gluten-containing products was assessed by a combination of self-reported adherence and coeliac disease serology. Participants were rated strictly adherent if they reported being on a strict gluten-free diet in dietary interview and were found to be negative for serum endomysial (EmA) and transglutaminase 2 (TG2-ab) antibodies.

Serum IgA class EmA were measured by an indirect immunofluorescence method with human umbilical cord as substrate [22] and serum IgA class TG2-ab by enzyme-linked immunosorbent assay (QUANTA Lite h-tTG IgA, INOVA Diagnostics, San Diego, CA, USA). A dilution of 1: ≥5 for EmA was considered positive and positive samples were further diluted 1:50, 1:100, 1:200, 1:500, 1:1000, 1:2000 and 1:4000. In TG2-ab unit values ≥30 U/I were considered positive. In cases known to involve selective IgA deficiency, the corresponding IgG-class antibodies were applied.

### Statistics

Categorical data were described using percentages and quantitative data using medians with range. Binary logistic regression analysis was used to identify factors associated with reduced quality of life. Results are presented as odds ratios (OR) with 95% confidence intervals (CI). A p-value ≤ 0.05 was considered statistically significant. *T*-test was used to compare the GSRS and PGWB mean scores between coeliac patients and healthy controls.

If a patient failed to answer on one or two items in the PGWB or GSRS questionnaire, the missing answer was replaced by the mean value of the other scores for the same subject. If more than two answers were missing the questionnaire was disqualified. All statistical data were analyzed by the Predictive Analytic SoftWare for Windows version 19 (IBM Corporation, Armonk, NY, USA).

## Results

Clinical characteristics of the study cohort are shown in Table 1. In total, 69% of the subjects were diagnosed on the basis of classical gastrointestinal presentation and 31% on the basis of extraintestinal symptoms or by screening in at-risk coeliac disease groups. Altogether 88% of the subjects were strictly adherent; none reported totally unrestricted gluten consumption and 82% used oats as a regular part of their diet. Lactose intolerance was present in 17%, food allergy in 5% and fructose intolerance in none of the participants. Further, 6% of the subjects had reflux disease, 4% gastritis, 2% diverticulosis and 2% cholelithiasis. The most common psychiatric disorder was depression, with a prevalence of 2% (78% of subjects with psychiatric diagnosis).

**Table 1:** Demographic data and clinical characteristics of the 596 coeliac disease patients

**Female, n (%)**	**452 (76)**
Current age, median (range), years	55 (19–92)
Age at diagnosis, median (range), years	44 (1–82)
Site of diagnosis, n (%)	
Primary care	149 (25)
Secondary or tertiary care	446 (75)
Presenting symptom at time of diagnosis, n (%)	
Gastrointestinal ^1^	410 (69)
Extraintestinal ^2^	95 (16)
Screen-detected ^3^	91 (15)
Coeliac disease in family, n (%)	372 (62)
Coeliac disease-associated disorders, n (%)	
Type 1 diabetes mellitus	14 (2)
Thyroid disease	107 (18)
Gluten-free diet, n (%)	
Strict	520 (88)
Occasional gluten	76 (12)
No diet	0
Consumption of oats, n (%)	487 (82)
Duration of gluten-free diet, median (range), years	10 (1–53)

^1^Stomach pain, diarrhoea, constipation, heartburn, flatulence, swelling, anaemia, malabsorption.

^2^Dermatitis herpetiformis, tiredness, joint pains, neurologic symptoms, gynaecologic problems.

^3^Coeliac disease in relatives, presence of autoimmune disorder.

The mean total GSRS score was 2.1 (range 1.0- 4.8) in the study group and 1.9 (range 1.0- 4.2) in the controls. The score was higher than 1 SD compared to the control mean in 25% of the participants. There was no significant association between the persistent gastrointestinal symptoms and gender or current age, but patients diagnosed between the age of 25 and 60 years had more persistent symptoms than those diagnosed at younger or older age (Table 2). Further, subjects who had suffered from moderate or severe symptoms at the time of the diagnosis or had had symptoms more than 10 years before it had more current gastrointestinal symptoms than initially asymptomatic patients. Patients diagnosed on extraintestinal symptoms had fewer current symptoms than those with gastrointestinal presentation (Table 2). The presence of thyroid disease, non-coeliac food intolerance, other gastrointestinal disease and any coeliac disease-related co-morbidity also increased the risk of persistent symptoms. None of the other variables examined, including dietary non-adherence and consumption of oats, was associated with persistent symptoms. The GSRS mean total score was significantly higher in the study participants than in control subjects (p= 0.003).

**Table 2:** Factors associated with persistent gastrointestinal symptoms* **in treated coeliac disease patients**

**Variable**		**n**	**Symptoms, %**	**OR**	**95% CI**	**p-value**
Gender	Male	144	22	1.00		
	Female	452	26	1.26	0.80-1.97	0.317
Present age, years	< 25	12	20	1.00		
	25-60	380	24	1.25	0.26-6.01	0.778
	> 60	204	27	1.51	0.31-7.33	0.611
Age at diagnosis, years	< 25	79	13	1.00		
	25-60	457	27	2.86	1.38-5.91	0.050
	> 60	60	20	1.99	0.77-5.10	0.154
Symptoms at diagnosis	Gastrointestinal	410	27	1.00		
	Extraintestinal	95	17	0.56	0.31-1.00	0.050
	Screen-detected	91	24	0.88	0.52-1.49	0.636
Duration of symptoms before diagnosis, years	No symptoms	37	11	1.00		
	≤ 10	350	22	2.33	0.80-6.77	0.121
	> 10	182	34	4.16	1.41-12.28	0.010
Severity of symptoms at diagnosis	No symptoms	62	13	1.00		
	Moderate	457	25	2.27	1.05-4.91	0.037
	Severe	77	31	3.06	1.26-7.41	0.013
Site of diagnosis	Hospital	446	25	1.00		
	Primary care	149	25	1.01	0.66-1.55	0.967
Coeliac disease in family	Yes	372	24	1.00		
	No	224	25	1.02	0.73-1.57	0.731
Type 1 diabetes mellitus	No	582	25	1.00		
	Yes	14	29	1.23	0.38-3.97	0.735
Thyroid disease	No	489	22	1.00		
	Yes	107	36	2.02	1.29-3.16	0.002
Malignancy	No	565	25	1.00		
	Yes	31	26	1.04	0.47-2.43	0.884
Psychiatric disease	No	578	24	1.00		
	Yes	18	39	1.99	0.76-5.22	0.164
Neurologic disease	No	520	24	1.00		
	Yes	76	30	1.38	0.81-2.35	0.231
Other food intolerance	No	470	22	1.00		
	Yes	126	33	1.74	1.13-2.67	0.012
Other gastrointestinal disease	No	388	22	1.00		
	Yes	208	34	1.80	1.16-2.80	0.009
Any coeliac disease-related co-morbidity	No	72	14	1.00		
	Yes	523	26	2.20	1.10-4.40	0.027
Strict gluten-free diet	Yes	523	24	1.00		
	No	73	27	1.17	0.68-2.04	0.570
Consumption of oats	No	105	25	1.00		
	Yes	487	23	0.87	0.54-1.41	0.568
Duration of diet, years	> 10	270	27	1.00		
	5-10	195	23	0.80	0.51-1.25	0.326
	<5	131	25	0.92	0.57-1.42	0.730
Professional dietary advice	Yes	484	24	1.00		
	No	112	27	1.15	0.72-1.83	0.564
Regular follow-up	Yes	166	25	1.00		
	No	412	23	0.93	0.61-1.41	0.721

*Defined as a score >1 standard deviation from the mean Gastrointestinal Symptom Rating Scale total score of healthy controls; CI, confidence interval; OR, odds ratio defined by bivariate logistic regression.

The mean PGWB total score was 102.8 (range 29.0-132.0) in the study group and 105.3 (range 65.0-126.0) in the controls. Altogether 25% of the participants had the score lower than 1 SD compared to the control mean. Reduced health-related quality of life was seen in patients with manifest symptoms prior to diagnosis compared with asymptomatic subjects; the difference was evident in particular among those with symptoms for more than 10 years. Further, subjects with any psychiatric co-morbidity had reduced quality of life (Table 3). A similar trend was observed in patients with neurological disease and gastrointestinal co-morbidity, but the results in question were not statistically significant (Table 3). Of note, patients with persistent gastrointestinal symptoms were also more likely to have reduced quality of life (OR 4.66, CI 3.01-7.00, p < 0.001). The coeliac patients had lower mean PGWB scores than healthy subjects (p = 0.05).

**Table 3:** Factors associated with persistently reduced quality of life* **in treated coeliac disease patients**

**Variable**		**n**	**Reduced, %**	**OR**	**95% CI**	**p-value**
Gender	Male	144	24	1.00		
	Female	452	25	1.07	0.69-1.67	0.777
Present age, years	< 25	12	10	1.00		
	25-60	380	26	3.21	0.40-25.66	0.272
	> 60	204	21	2.40	0.30-19.51	0.413
Age at diagnosis, years	< 25	79	25	1.00		
	25-60	457	25	0.97	0.56-1.68	0.911
	> 60	60	22	0.82	0.37-1.81	0.617
Symptoms at diagnosis	Gastrointestinal	410	26	1.00		
	Extraintestinal	95	19	0.65	0.37-1.14	0.136
	Screen-detected	91	22	0.79	0.46-1.36	0.389
Duration of symptoms before diagnosis, years	No symptoms	37	8	1.00		
	≤ 10	350	24	3.58	1.07-11.95	0.038
	> 10	182	28	4.41	1.30-15.01	0.017
Severity of symptoms at diagnosis	No symptoms	62	18	1.00		
	Moderate	457	26	1.61	0.81-3.20	0.170
	Severe	77	22	1.31	0.56-3.06	0.527
Site of diagnosis	Hospital	446	25	1.00		
	Primary care	149	24	0.97	0.63-1.50	0.902
Coeliac disease in family	Yes	372	25	1.00		
	No	224	24	0.97	0.66-1.42	0.864
Type 1 diabetes mellitus	No	582	25	1.00		
	Yes	14	21	0.84	0.23-3.04	0.785
Thyroid disease	No	489	24	1.00		
	Yes	107	25	1.05	0.65-1.70	0.845
Malignancy	No	565	25	1.00		
	Yes	31	13	0.44	0.15-1.28	0.132
Psychiatric disease	No	578	23	1.00		
	Yes	18	67	6.63	2.44-17.99	<0.001
Neurologic disease	No	520	23	1.00		
	Yes	76	33	1.62	0.96-2.72	0.070
Other food intolerance	No	470	24	1.00		
	Yes	126	28	1.24	0.80-1.94	0.335
Other gastrointestinal disease	No	388	23	1.00		
	Yes	208	32	1.56	1.00-2.45	0.052
Any coeliac disease-related co-morbidity	No	72	26	1.00		
	Yes	523	24	0.89	0.51-1.56	0.691
Strict gluten-free diet	Yes	523	24	1.00		
	No	73	27	1.19	0.69-2.07	0.539
Consumption of oats	No	105	22	1.00		
	Yes	487	25	1.19	0.72-1.98	0.497
Duration of diet, years	> 10	270	23	1.00		
	5-10	195	23	0.96	0.61-1.52	0.875
	<5	131	29	1.35	0.84-2.18	0.221
Professional dietary advice	Yes	484	24	1.00		
	No	112	28	1.23	0.77-1.95	0.385
Regular follow-up	Yes	166	24	1.00		
	No	412	24	1.00	0.65-1.52	0.986

*Defined as a score >1 standard deviation from the mean Psychological General Well-Being total score of healthy controls; CI, confidence interval; OR, odds ratio defined by bivariate logistic regression.

## Discussion

The results of our nationwide study showed that up to 25% of adult coeliac disease patients suffer from persistent gastrointestinal symptoms despite a strict gluten-free diet. These findings show an excess of gastrointestinal complaints in treated coeliac patients, although to a somewhat lesser extent than noted in some previous studies [23,24]. Yet it needs to be remembered that comparison between studies is difficult because both the methods to measure and definitions used for persistent symptoms may differ. Further, it is important to distinguish primary non-responsive patients from those investigated in the present study; common reasons for non-responsive coeliac disease are inadvertent gluten intake, refractory coeliac disease and concomitant diseases such as malignancies, microscopic colitis and pancreatic insufficiency [5,25,26]. Patients with any such concomitant condition or refractory coeliac disease were excluded from the present study. Furthermore, although no histological evaluation was undertaken here, judging from both dietary interview and serological testing the majority of participants were adhering strictly to the diet. It would thus seem unlikely that inadvertent gluten intake or presence of the aforesaid adjunct disease would explain the ongoing gastrointestinal symptoms.

In the present study, coeliac patients diagnosed at working age had more current symptoms than those diagnosed at an either younger or older age. Although we cannot confirm the reason for this age dependency of the symptoms, it is possible that changes in established dietary habits are more difficult to cope with simultaneously with other challenges encountered in daily work and family life. Likewise, for some unexplained reason, irritable bowel syndrome (IBS) often emerges in early adulthood [27]. Interestingly, also severe and long-lasting symptoms prior to diagnosis predispose coeliac patients to persistent symptoms. It has been suggested that such long-lasting abdominal complaints result in a chronic cycle of pain in consequence of changes in the gut-brain axis [28,29]. Of note, similar chronic alterations have been hypothesized to be involved in the pathogenesis of IBS, which often co-exists with coeliac disease [30-32]. There is also evidence suggesting that the symptoms in IBS might be caused by continuous low-grade small-bowel mucosal inflammation [30,31,33], a condition which may persist in coeliac disease despite a strict gluten-free diet [34,35]. Finally, small-intestinal bacterial overgrowth may account for symptoms in both coeliac disease and IBS and is also accompanied by mucosal inflammation [33,36,37]. Whatever mechanisms lie behind the persistent symptoms, our results indicate that they could be ameliorated by diagnosing coeliac disease as early as possible.

The results obtained showed the presence of a coeliac disease-related co-morbidity, another gastrointestinal disorder or non-coeliac food intolerance to predispose to persistent symptoms. It is possible that concomitant gastrointestinal co-morbidity further reinforces the impact of the aforementioned long-lasting changes in the gut-brain axis. Interestingly, a particular association was seen between persistent symptoms and concomitant thyroid disease, which when untreated is known to may cause abdominal complaints, these usually recovering on treatment [38]. However, some patients evince persistent clinical symptoms and reduced health-related quality of life even while on treatment and euthyroid [39]. The pathogenic mechanisms underlying this remain unclear, but clearly the possible connection between thyroid disease and persistent gastrointestinal symptoms in coeliac disease calls for further investigation.

As was the case with gastrointestinal symptoms, a substantial proportion of the coeliac patients here showed reduced health-related quality of life while on a gluten-free diet. Again, long duration of symptoms before diagnosis was a predisposing factor. In contrast, there was no association between clinical manifestations of coeliac disease at diagnosis and current quality of life. This is in line with our previous results showing similar health-related quality of life in screen-detected and symptom-detected coeliac patients on a gluten-free diet [12,13]. These findings constitute further evidence that early detection and dietary treatment of coeliac disease is beneficial even in screen-detected patients with mild or atypical symptoms at diagnosis.

There was a significant association between reduced quality of life and the presence of psychiatric co-morbidities in our study patients. This is in accord with some previous findings and there is also evidence that such coeliac patients might benefit from intensified psychological counseling [8,18,40-42]. Consequently, health-care professionals should pay particular attention to this patient group, who may need special support with their dietary treatment [42]. Since in the present study 78% of participants with a psychiatric disorder suffered from depression, the role of other psychiatric disorders needs further investigation. Although not statistically significant, there was a trend suggesting that the presence of certain other co-morbidities, for example neurological disorders, other gastrointestinal diseases or non-coeliac food intolerance are factors predisposing to reduced quality of life in coeliac disease. Further studies are warranted to clarify the significance of these issues.

A major strength of our study was a large study cohort with well-defined coeliac disease diagnoses and clinical data. In addition, there were a substantial number of screen-detected patients and subjects with extraintestinal presentation of coeliac disease. A possible limitation, on the other hand, was that a substantial proportion of the participants were recruited through local or national coeliac disease associations, which might have caused some selection bias. Furthermore, our participants showed excellent adherence to the gluten-free diet, this however being in accord with our earlier findings [12]. Although good adherence is beneficial for the patient, in this study it might have concealed the association between poor adherence and reduced quality of life. As a consequence, results may be different in countries where adherence to a gluten-free diet is lower. Although the threshold value of 1 SD to define increased symptoms and reduced quality of life has also been used in other studies, it is somewhat artificial. The fact that healthy controls were not precisely matched with the study participants was also a limitation.

The majority of participants used oats as a regular part of their diet, and this was not associated with increased gastrointestinal symptoms or reduced quality of life. This is important, since the use of oat-containing products in coeliac disease has hitherto remained controversial [43]. Based on our previous results [44] and the findings here, purified oats could be a regular part of a gluten-free diet. One important issue which should be considered in evaluating gastrointestinal symptoms in coeliac patients is that commercial gluten-free products may have marked qualitative differences, for example in the use of additives and preservatives and the amount of fibre [45].

## Conclusions

As a conclusion, we showed that many coeliac disease patients suffer from persistent gastrointestinal symptoms and reduced quality of life despite strict dietary treatment. In particular, long-lasting and severe symptoms before the diagnosis and concomitant thyroid, gastrointestinal and psychiatric co-morbidities were significant risk factors for these ongoing health concerns. The results emphasize the importance of early diagnosis and careful follow-up in coeliac disease.

## Competing interests

The authors declare that they have no competing interests.

## Authors’ contributions

PP: Study concept and design; analysis and interpretation of data; statistical analysis; writing of the manuscript. KK: Study concept and design; analysis and interpretation of data; statistical analysis; drafting of the manuscript; critical revision of the manuscript for important intellectual content. AU: Study concept and design; acquisition of data; critical revision of the manuscript for important intellectual content. PC: Study concept and design; acquisition of data; critical revision of the manuscript for important intellectual content. HH: Study concept and design; critical revision of the manuscript for important intellectual content; statistical analysis. MM: Study concept and design; acquisition of data; critical revision of the manuscript for important intellectual content. KK: Study concept and design; analysis and interpretation of data; statistical analysis; drafting of the manuscript; critical revision of the manuscript for important intellectual content. All authors read and approved the final manuscript.

## Pre-publication history

The pre-publication history for this paper can be accessed here:

http://www.biomedcentral.com/1471-230X/13/75/prepub
